# Cancer Patients Enrolled in a Smoking Cessation Clinical Trial: Characteristics and Correlates of Smoking Rate and Nicotine Dependence

**DOI:** 10.1155/2018/2438161

**Published:** 2018-02-26

**Authors:** Andrew Miele, Morgan Thompson, Nancy C. Jao, Ravi Kalhan, Frank Leone, Lee Hogarth, Brian Hitsman, Robert Schnoll

**Affiliations:** ^1^Department of Psychiatry, University of Pennsylvania, Philadelphia, PA, USA; ^2^Department of Preventive Medicine, Northwestern University Feinberg School of Medicine, Chicago, IL, USA; ^3^Robert H. Lurie Comprehensive Cancer Center, Northwestern University, Chicago, IL, USA; ^4^Department of Medicine, Northwestern University, Evanston, IL, USA; ^5^Pulmonary, Allergy & Critical Care Division, University of Pennsylvania, Philadelphia, PA, USA; ^6^School of Psychology, University of Exeter, Exeter, UK

## Abstract

**Introduction:**

A substantial proportion of cancer patients continue to smoke after their diagnosis but few studies have evaluated correlates of nicotine dependence and smoking rate in this population, which could help guide smoking cessation interventions.

**Aim:**

This study evaluated correlates of smoking rate and nicotine dependence among 207 cancer patients.

**Methods:**

A cross-sectional analysis using multiple linear regression evaluated disease, demographic, affective, and tobacco-seeking correlates of smoking rate and nicotine dependence. Smoking rate was assessed using a timeline follow-back method. The Fagerström Test for Nicotine Dependence measured levels of nicotine dependence.

**Results:**

A multiple linear regression predicting nicotine dependence showed an association with smoking to alleviate a sense of addiction from the Reasons for Smoking scale and tobacco-seeking behavior from the concurrent choice task (*p* < .05), but not with affect measured by the HADS and PANAS (*p* > .05). Multiple linear regression predicting prequit showed an association with smoking to alleviate addiction (*p* < .05). ANOVA showed that Caucasian participants reported greater rates of smoking compared to other races.

**Conclusions:**

The results suggest that behavioral smoking cessation interventions that focus on helping patients to manage tobacco-seeking behavior, rather than mood management interventions, could help cancer patients quit smoking.

## 1. Introduction

The US Surgeon General concluded that a causal relationship exists between continued tobacco use among cancer patients and poor clinical outcomes, including cancer-specific mortality, an increased risk of disease recurrence, and decreased response to cancer treatment [1]. Despite these risks, upwards of 50% of those who were smokers when diagnosed continue to smoke or relapse soon after receiving their diagnosis [2, 3]. Identifying effective smoking cessation interventions for cancer patients is therefore a priority [4]. This requires identifying correlates of smoking behavior, which can serve as intervention targets [2].

There has been little consistency in linking disease-related characteristics (e.g., cancer type, stage) and demographic variables (e.g., sex, race) to cancer patient smoking rate [5]. Some studies have found that medical variables (cancer site and cancer type), and certain demographic variables [1, 6], are related to smoking rate and smoking cessation among cancer patients [7]. But other studies have failed to demonstrate a relationship between disease-related characteristics, demographic variables, and cancer patient smoking rate [8]. In addition, negative affect [9–11] and nicotine withdrawal and craving [12, 13] have been associated with a greater smoking frequency or smoking relapse in this population. This literature provides little consensus as to the key predictors of smoking frequency and dependence in cancer patients, which the current study will address by examining a range of possible predictors.

Recently, Hogarth and colleagues validated a concurrent choice task as an index of the relative reward value that individuals ascribe to tobacco. In this task, participants make forced choices (over successive trials) between a response that produces tobacco and a response that produced food. In the general population, preferential choice of tobacco over food is associated with smoking frequency, dependence severity, and subject craving and can be modulated by smoking abstinence, smoking satiety, nicotine replacement medication, negative mood induction, and smoking related stimuli [14–20]. The current study tested whether smoking frequency and tobacco dependence severity in cancer patients would be associated with greater relative reward value ascribed to tobacco in the concurrent choice task. This finding would suggest that smoking in cancer patients is an economic decision driven by the greater relative value of tobacco.

A better understanding of key correlates of smoking and dependence in cancer patients could guide intervention development. Given that negative affect has been reliably associated with smoking in cancer patients [10, 11], we expect to replicate this association here. We also expect to demonstrate a novel association between tobacco choice and smoking/dependence in cancer patients. These findings would suggest that smoking cessation interventions for cancer patients should integrate mood management with treatment strategies derived from economic decision theory [21].

## 2. Methods

### 2.1. Participants

This study, which represents secondary analyses, utilized data from a National Cancer Institute (NCI) smoking cessation clinical trial (i.e., a parent trial; clinicaltrials.gov: NCT01756885) comparing 12 versus 24 weeks of varenicline and behavioral counseling. Participants were recruited from cancer centers in Philadelphia and Chicago using proactive recruitment within the electronic medical record (EMR) and clinician referrals. To be eligible, for the parent trial and for this study, participants had to be ≥18 years old, have a cancer diagnosis within the past 5 years, report smoking ≥ 5 cigarettes/week, be interested in quitting (assessed using self-reported response to “Are you interested in quitting smoking?” during phone screen), and have a Karnofsky score (functional capacity) > 50 or an Eastern Cooperative Oncology Group (ECOG) performance status (disease progression) ≤ 2. All participants were either actively in medical treatment for a cancer diagnosis or in follow-up for a diagnosis within the past 5 years, which is consistent with definitions of cancer patients when the trial began recruitment. Exclusions for this study, which were the same as those for the parent trial, can be found in a previous study [22]. Following telephone initial eligibility screening, participants were invited to an in-person intake session to determine final eligibility. Of the 569 participants eligible at phone screen, 282 were eligible at intake and 75 refused to participate, leaving a sample of 207 participants (recruitment rate = 73.4%). Among eligible participants who declined to enroll, the primary reasons given for refusal were lack of interest in the study, time commitment, and reluctance to take the study medication. No significant differences were found between those who chose to participate and those who declined participation in terms of demographic, smoking related, and disease characteristics. All participants who attended their intake session received $10 for travel, plus an additional $10 for time, if the entire session was completed successfully.

### 2.2. Procedures

Written informed consent was obtained at an intake session and those eligible were randomized to 12 or 24 weeks of varenicline, with 5 counseling sessions. Data from the present analyses were taken from participants prior to completing their first counseling session and initiating treatment. This included assessment of smoking using timeline follow-back, questionnaires, and a computer-based, concurrent choice task that measured tobacco-seeking behavior.

### 2.3. Measures

#### 2.3.1. Demographics, Disease Characteristics, and Smoking

Participants self-reported demographics and smoking rate and completed the Fagerström Test for Nicotine Dependence (FTND) [23]. Disease characteristics (e.g., tumor site) were ascertained from the EMR.

#### 2.3.2. Affect

The Positive and Negative Affect Schedule [24] assessed positive (e.g., enthusiastic) and negative (e.g., distressed) affect. The Hospital Anxiety and Depression Scale [25] assessed depression and anxiety symptoms. The Reasons for Smoking (RFS) scale assessed motivations for smoking (i.e., smoking to reduce negative affect, provide stimulation, or alleviate addiction) [26].

#### 2.3.3. Withdrawal and Craving

The Shiffman-Jarvik Withdrawal Scale (SJWS) assessed nicotine withdrawal (i.e., craving, stimulation/sedation, psychological symptoms, and appetite) [27]. The Brief Questionnaire of Smoking Urges (QSU-b) [28] assessed craving. Five of the items measured the desire to smoke for reward (Factor 1) and the other five measured the need to smoke for negative affect relief (Factor 2). The sum of items 1–10 forms the QSU-b total.

#### 2.3.4. Concurrent Choice Task

This computer-based task assessed preferential tobacco versus food choice [14]. Participants pressed the left or right key to produce an image of smoking versus chocolate, respectively (the response-reward contingencies were counterbalanced between subjects). This measure is an analogue of animal choice models [29] and simplifies earlier human tobacco choice procedures [30]. Outcomes were percent tobacco versus chocolate choice to index the relative tobacco value and overall reaction time of choices as a crude index of cognitive function.

### 2.4. Data Analysis

The characteristics of the sample, including demographic, disease-related, and smoking data, were summarized using descriptive statistics (e.g., means, standard deviations). Univariate statistics (Pearson correlation and ANOVA) were used to examine if variables in Table 1 were associated with smoking rate (self-reported number of cigarettes in the past 7 days) and FTND. Variables associated with smoking rate or FTND (*p* < .05) from these analyses were included in separate multiple linear regression models in order to focus on them as distinct measures of smoking behavior. Predictors of smoking rate and FTND within models were evaluated using 95% confidence intervals and probability values. Predictors were simultaneously entered into respective linear regression models and categorical variables were dummy coded. These procedures are similar to those used in past studies [10].

## 3. Results

### 3.1. Sample Characteristics

Participant characteristics are listed in Table 1. More than half of the sample was female, more than a third were from ethnic/racial minority groups, and close to two-thirds did not have a college degree. Close to one-fifth of the sample had head and neck or lung cancer and ~60% had undergone medical treatment in the past 30 days. Overall, participants averaged smoking 15 cigarettes/day for >40 years. Average pack year was calculated for the population by multiplying the number of packs smoked per day by the number of years of smoking. The average pack year for the sample population was 30.70 (SD = 20.60).

### 3.2. Univariate Correlates of Smoking Rate and FTND

Table 2 outlines the Pearson correlations between demographic, medical, and smoking variables listed in Table 1. A higher smoking rate was associated with smoking to alleviate addiction (*r* = .36, *p* < .001), physical symptoms (*r* = .266, *p* < .001), and nonphysiological symptoms (*r* = .18, *p* < .05) and provide stimulation (*r* = .19, *p* < .01), on the RFS. A higher level of nicotine dependence was associated with higher withdrawal on the SJWS (*r* = .29, *p* < .001) and craving on the QSU-b (*r* = .32, *p* < .001), including both QSU-B subscales measuring smoking urges associated with pleasure (Factor 1) (*r* = .40, *p* < .001) and reducing negative affect (Factor 2) (*r* = .25, *p* < .001). On the RFS, higher levels of nicotine dependence were associated with smoking to alleviate negative affect (*r* = .23, *p* ≤ .001), addiction (*r* = .40, *p* < .001), and physiological symptoms (*r* = .40, *p* < .001) as well as nonphysiological symptoms (*r* = .27, *p* < .001) and for stimulation (*r* = .34, *p* < .001). Higher nicotine dependence was associated with a preference for tobacco on the choice task (*r* = .28, *p* < .05). Caucasians and participants with tumors other than lung or head and neck reported a higher smoking rate when compared to patients of racial minority groups and patients with lung or head and neck cancer (*F*[1,202] = 16.26, *p* < .001; *F*[1,201] = 4.66, *p* = .032, resp.). Participants who were unemployed had higher levels of nicotine dependence compared to those who were employed (*F*[1,201] = 5.77, *p* = .017). Study site was not associated with outcomes.

### 3.3. Multivariate Correlates of Smoking Rate and FTND

Table 3 shows the results of a multivariate linear regression for smoking rate. Caucasians reported a higher smoking rate. Smoking to alleviate addiction was also associated with higher smoking rates.  Table 4 shows the results of a multiple linear regression for severity of nicotine dependence. Smoking to alleviate addiction and a preference for tobacco on the choice task were associated with a higher level of nicotine dependence.

## 4. Discussion

This study analyzed demographic, disease, affective, and tobacco-seeking correlates of smoking rate and nicotine dependence among cancer patients to identify potential targets for smoking cessation interventions. This study is the first to employ a novel concurrent choice task, designed to measure tobacco-seeking behavior, with cancer patients. Our results suggest that nicotine dependence in cancer patients is associated with smoking to alleviate a sense of addiction and with the greater relative value of tobacco over natural rewards indexed by the choice task. These findings suggest that treatments for cancer patients might focus on developing coping skills for addiction or on increasing engagement with nonsmoking rewards through contingency management or behavioral activation. The lack of association between nicotine dependence and smoking to alleviate negative affect suggests that mood management treatments may be less effective.

In the final models, only race and cancer site were correlated with smoking behavior. The association between race and smoking rate is consistent with studies in the general population that show African Americans, Asian/Pacific Islanders, and Hispanic/Latinos to be more likely to be light smokers, versus Caucasian smokers [31]. As such, in the oncologic context, it may be necessary to consider the literature on smoking cessation interventions across light versus heavy smokers to identify the most suitable interventions for racial/ethnic minority groups. Contrary to previous findings with cancer patients [9–11], affective measures were not associated with smoking behavior, although this may be related to patients with psychiatric comorbidity not enrolling in the trial. In contrast, the RFS subscale focused on alleviating addiction was associated with smoking rate and nicotine dependence, and the concurrent choice task was associated with dependence. Consistent with recent models of tobacco dependence [32], these results suggest treatments for cancer patients should focus on training skills that counteract the belief that smoking helps cope with adverse states [33] and focus on increasing the relative value of nonsmoking rewards through contingency management or behavioral activation [21].

This study is limited by the use of cross-sectional data and participants enrolled in a clinical trial. Further, while the results indicate that smoking behavior among cancer patients is related to coping with sense of addiction and greater value ascribed to tobacco, rather than demographic, disease-related, or affective factors, these results may not be unique to cancer patients compared to the general population.

## Figures and Tables

**Table 1:** Characteristics of program enrollees (*N* = 207).

Characteristic	Enrollees
	*N* (%) or mean (SD)
*Demographic variables*	
Gender	
Male	102 (49.3)
Female	105 (50.7)
Education^*∗*^	
Below college grad	132 (63.8)
College grad or beyond	73 (35.3)
Marital status	
Not married	102 (49.3)
Married	103 (49.8)
Employment status^*∗*^	
Employed	100 (48.3)
Not employed	105 (50.7)
Income^*∗*^	
<20,000	44 (21.3)
20,000 to 75,000	91 (44)
75,000<	69 (33.3)
Race	
Caucasian	142 (68.6)
Non-Caucasian	65 (31.4)
Age	58.5 (9.4)
*Medical variables*	
Tumor site^*∗*^	
Head and neck or lung	39 (18.8)
Other sites	167 (80.7)
Tumor stage	
Stages 0–2	41 (19.8)
Stages 3-4	45 (21.7)
Remission/stage not specified	121 (58.5)
Time from diagnosis to intake (days)^*∗*^	102.7 (19.6)
Cancer treatment in last month^*∗*^	
Radiation	21 (10.1)
Chemotherapy	42 (20.3)
Surgery	25 (12.1)
Hormone therapy	22 (10.6)
Radiation and chemotherapy	12 (5.8)
Chemotherapy and hormone therapy	5 (2.4)
*Smoking variables*	
Age smoking was started	16.7 (5.1)
Pack years	30.70 (20.60)
QSU-b^⋀^	
Factor 1	16.7 (8.56)
Factor 2	8.05 (5.16)
Total	27.46 (14.01)
SJWS	
Craving	15.69 (7.45)
Physical	4.36 (2.16)
Stimulation	2.14 (1.53)
Psychological	11.75 (4.29)
Appetite	1.99 (1.55)
RFS	
Addiction	4.58 (2.16)
Stimulation	4.68 (3.11)
Negative affect	5.03 (2.47)
Physiological	8.25 (4.20)
Not physiological	6.02 (3.03)
Number of cigarettes smoked 7 days prior to PQ^*∗*^	75.81 (49.19)
FTND score^*∗*^	4.5 (2.1)
Concurrent choice task (% of choice tobacco versus chocolate)^*∗*^	27.99 (27.12)
*Affective variables*	
PANAS	
Positive affect	35.51 (7.82)
Negative affect	13.72 (4.28)
HADS	
Anxiety	4.05 (2.82)
Depression	2.45 (2.44)

*Note*. *∗* indicates missing data (<5%); QSU-b: Brief Questionnaire of Smoking Urges; ⋀ = see measures for description of Factors 1 and 2; SJWS = Shiffman-Jarvik Withdrawal Scale; RFS = Reasons for Smoking; PQ = prequit visit; FTND = Fagerström Test for Nicotine Dependence; PANAS = the Positive and Negative Affect Schedule; HADS = the Hospital Anxiety and Depression Scale.

**Table 2:** Pearson correlation analyses of demographic, medical, and smoking variables from Table 1.

	1	2	3	4	5	6	7	8	9	10	11	12	13	14	15	16	17	18	19	20	21	22	23	24	25
(1) Income	1																								
(2) Age	.03	1																							
(3) Time from dx to intake	−.11	−.08	1																						
(4) Age smoking was started	.12	.02	−.10	1																					
(5) Pack years	.13	.35^*∗∗*^	.04	−.24^*∗∗*^	1																				
(6) QSU-b Factor 1	−.15^*∗*^	−.22^*∗∗*^	−.02	−.21^*∗∗*^	.11	1																			
(7) QSU-b Factor 2	−.13	−.11	−.06	−.07	.06	.66^*∗∗*^	1																		
(8) QSU-b total	−.16^*∗*^	−.18^*∗∗*^	−.04	−.18^*∗*^	.10	.95^*∗∗*^	.86^*∗∗*^	1																	
(9) SJWS craving	−.06	−.15^*∗*^	−.05	−.11	.12	.82^*∗∗*^	.62^*∗∗*^	.81^*∗∗*^	1																
(10) SJWS physical	−.06	−.16^*∗*^	−.11	−.07	−.04	.32^*∗∗*^	.38^*∗∗*^	.37^*∗∗*^	.40^*∗∗*^	1															
(11) SJWS stimulation	−.01	−.03	−.03	−.03	.01	.10	.10	.10	.24^*∗∗*^	.26^*∗∗*^	1														
(12) SJWS psychological	.05	−.01	.01	−.11	.09	.12	.23^*∗∗*^	.18^*∗*^	.21^*∗∗*^	.35^*∗∗*^	.68^*∗∗*^	1													
(13) SJWS appetite	−.17^*∗*^	.00	−.07	−.08	−.10	.10	.07	.11	.11	.20^*∗∗*^	.12	.15^*∗*^	1												
(14) RFS addiction	.01	−.20^*∗∗*^	.07	−.20^*∗∗*^	.30^*∗∗*^	.36^*∗∗*^	.25^*∗∗*^	.33^*∗∗*^	.31^*∗∗*^	.11	.00	.17^*∗*^	−.05	1											
(15) RFS stimulation	.07	−.15^*∗*^	−.04	−.17^*∗*^	.24^*∗∗*^	.38^*∗∗*^	.34^*∗∗*^	.39^*∗∗*^	.34^*∗∗*^	.17^*∗*^	.09	.23^*∗*^	−.01	.63^*∗∗*^	1										
(16) RFS negative affect	−.00	−.30^*∗∗*^	.10	−.20^*∗∗*^	.06	.23^*∗∗*^	.22^*∗∗*^	.25^*∗∗*^	.19^*∗∗*^	.09	.03	.15^*∗*^	−.04	.61^*∗∗*^	.58^*∗∗*^	1									
(17) RFS physiological	.05	−.19^*∗∗*^	−.01	−.21^*∗∗*^	.29^*∗∗*^	.41^*∗∗*^	.32^*∗∗*^	.40^*∗∗*^	.37^*∗∗*^	.18^*∗*^	.07	.24^*∗*^	−.02	.80^*∗∗*^	.95^*∗∗*^	.64^*∗∗*^	1								
(18) RFS not physiological	.01	−.27^*∗∗*^	.10	−.19^*∗∗*^	.11	.27^*∗∗*^	.25^*∗∗*^	.28^*∗∗*^	.22^*∗∗*^	.07	.03	.15^*∗*^	−.04	.75^*∗∗*^	.62^*∗∗*^	.95^*∗∗*^	.68^*∗∗*^	1							
(19) CPD 7 days prior to prequit visit	.12	−.07	.11	−.10	.71^*∗∗*^	.12	.05	.09	.12	.03	−.03	.00	−.12	.36^*∗∗*^	.19^*∗∗*^	.10	.27^*∗∗*^	.18^*∗*^	1						
(20) FTND score	−.14^*∗*^	−.13	.07	−.16^*∗*^	.49^*∗∗*^	.34^*∗∗*^	.25^*∗∗*^	.32^*∗∗*^	.29^*∗∗*^	.10	.02	.13	−.00	.40^*∗∗*^	.34^*∗∗*^	.23^*∗∗*^	.40^*∗∗*^	.27^*∗*^	.49^*∗∗*^	1					
(21) Choice task	−.13	−.07	.07	−.17^*∗*^	.14	.27^*∗∗*^	.26^*∗∗*^	.30^*∗∗*^	.18^*∗*^	.16^*∗*^	−.13	.01	.01	.17^*∗*^	.19^*∗*^	.22^*∗∗*^	.21^*∗∗*^	.21^*∗∗*^	.10	.28^*∗∗*^	1				
(22) PANAS positive affect	.14^*∗*^	−.10	.08	.11	−.03	−.07	−.12	−.09	−.12	−.13	−.44^*∗∗*^	−.48^*∗∗*^	−.08	−.04	−.07	−.07	−.07	−.06	.01	−.11	−.01	1			
(23) PANAS negative affect	−.05	−.06	−.04	−.01	−.03	.09	.13	.11	.08	.13	.18^*∗∗*^	.33^*∗∗*^	.09	.15^*∗*^	.23^*∗∗*^	.26^*∗∗*^	.23^*∗∗*^	.24^*∗∗*^	−.02	.11	.07	−.27^*∗∗*^	1		
(24) HADS anxiety	.06	−.05	.02	−.10	−.01	.07	.07	.07	.00	.11	.21^*∗∗*^	.26^*∗∗*^	.03	.15^*∗*^	.21^*∗∗*^	.28^*∗∗*^	.19^*∗∗*^	.28^*∗∗*^	.08	.04	−.05	−.30^*∗∗*^	.55^*∗∗*^	1	
(25) HADS depression	−.14	.03	−.02	−.10	.09	.12	.13	.14^*∗*^	.14^*∗*^	.06	.29^*∗∗*^	.24^*∗∗*^	.08	.10	.07	.12	.09	.11	−.00	.12	.02	−.55^*∗∗*^	.41^*∗∗*^	.48^*∗∗*^	1

*Note.* ^*∗*^ *p* < .05 and ^*∗∗*^*p* < .01; dx = diagnosis; CO = carbon monoxide (ppm); CPD = cigarettes per day; FTND = Fagerström Test for Nicotine Dependence. Since the outcomes (FTND and CPD) include smoking rate, pack year was not included in the models to reduce collinearity.

**Table 3:** Multiple regression analysis predicting number of cigarettes smoked one week before prequit visit.

Predictor variable	*B*	*t*	*p*	95% CI
RFS				
Addiction	.342	3.64	<.001	3.28 to 11.05
Stimulation	−.062	−.673	.50	−3.60 to 1.77
Cancer site	−.117	−1.62	.11	−31.0 to 3.09
Race	−.187	−2.49	.014	−32.92 to −3.81

*Note*. Model for cigarettes smoked before prequit visit: *F*(5,155) = 8.1; *p* < .001; RFS = Reasons for Smoking; SJWS = Shiffman-Jarvik Withdrawal Scale.

**Table 4:** Multiple regression analysis predicting scores on Fagerström Test for Nicotine Dependence.

Predictor variable	*B*	*t*	*p*	95% CI
Employment	.14	1.85	.07	−.383 to 4.02
Income	−.01	−.07	.95	−.495 to .463
Age smoking was started	−.09	−1.24	.22	−.112 to .026
RFS				
Negative affect	−.12	−1.24	.22	−.265 to .060
Addiction	.36	3.68	<.001	.165 to .548
Stimulation	.10	1.02	.31	−.066 to .207
QSU-b				
Factor 1	.17	1.24	.22	−.025 to .111
Factor 2	−.01	−.15	.88	−.084 to .072
SJWS craving	−.01	−.07	.94	−.075 to .070
% of choice (tobacco versus chocolate)	.16	2.05	.04	.000 to .025

*Note*. Model for level of nicotine dependence: *F*(10, 147) = 6.39; *p* < .001; RFS = Reasons for Smoking; QSU-b = Brief Questionnaire of Smoking Urges; SJWS = Shiffman-Jarvik Withdrawal Scale.
